# Cell Culture Isolation and Whole Genome Characterization of Hepatitis E Virus Strains from Wild Boars in Germany

**DOI:** 10.3390/microorganisms9112302

**Published:** 2021-11-05

**Authors:** Katja Schilling-Loeffler, Oliver Viera-Segura, Victor Max Corman, Julia Schneider, Ashish K. Gadicherla, Ulrich Schotte, Reimar Johne

**Affiliations:** 1 Department of Biological Safety, German Federal Institute for Risk Assessment, 10589 Berlin, Germany; Katja.Schilling-Loeffler@bfr.bund.de (K.S.-L.); Ashish.Gadicherla@bfr.bund.de (A.K.G.); 2 Laboratorio de Diagnóstico de Enfermedades Emergentes y Reemergentes, Departamento de Microbiología y Patología, Centro Universitario de Ciencias de la Salud, Universidad de Guadalajara, 44340 Guadalajara, Mexico; o.vierasegura@gmail.com; 3 Institute of Virology, Charité Universitätsmedizin Berlin, Corporate Member of Freie Universität Berlin, Humboldt-Universität zu Berlin, and Berlin Institute of Health, 10117 Berlin, Germany; victor.corman@charite.de (V.M.C.); julia.schneider@charite.de (J.S.); 4 German Centre for Infection Research (DZIF), Partner Site Charité, 10117 Berlin, Germany; 5 Central Institute of the Bundeswehr Medical Service Kiel, 24119 Kronshagen, Germany; UlrichSchotte@bundeswehr.org

**Keywords:** hepatitis E virus, wild boar, cell culture, hypervariable region, whole genome sequencing, zoonosis

## Abstract

Infection with hepatitis E virus (HEV) can cause acute and chronic hepatitis in humans. The HEV genotype 3 can be zoonotically transmitted from animals to humans, with wild boars representing an important reservoir species. Cell culture isolation of HEV is generally difficult and mainly described for human isolates so far. Here, five sera and five liver samples from HEV-RNA-positive wild boar samples were inoculated onto PLC/PRF/5 cells, incubated for 3 months and thereafter passaged for additional 6 weeks. As demonstrated by RT-qPCR, immunofluorescence and immune electron microscopy, virus was successfully isolated from two liver samples, which originally contained high HEV genome copy numbers. Both isolates showed slower growth than the culture-adapted HEV strain 47832c. In contrast to this strain, the isolated strains had no insertions in their hypervariable genome region. Next generation sequencing using an HEV sequence-enriched library enabled full genome sequencing. Strain Wb108/17 belongs to subtype 3f and strain Wb257/17 to a tentative novel subtype recently described in Italian wild boars. The results indicate that HEV can be successfully isolated in cell culture from wild boar samples containing high HEV genome copy numbers. The isolates may be used further to study the zoonotic potential of wild boar-derived HEV subtypes.

## 1. Introduction

The hepatitis E virus (HEV) is the causative agent of hepatitis E in humans [1]. This disease is mainly characterized as an acute hepatitis, but chronic infections in immunosuppressed transplant patients represent an increasing threat. In addition, extrahepatic disease manifestations have been described. In many European countries, the number of notified hepatitis E cases have markedly increased during the last years [2].

HEV is largely characterized as a non-enveloped particle containing a genome of a 7 kb long single-stranded RNA of positive polarity [3]. However, quasi-enveloped HEV particles have been recently demonstrated in patient serum and cell culture supernatant [4]. The genome contains the open reading frame (ORF)1 encoding a non-structural polyprotein, the ORF2 encoding the capsid protein, and the ORF3 encoding a small phosphoprotein [3].

The currently most relevant human-pathogenic HEVs are grouped into the genotypes (GT) 1–4 within the species *Orthohepevirus A* of the family *Hepeviridae* [3]. The GT1 and GT2 infect only humans and are mainly transmitted via contaminated drinking water. These genotypes have been responsible for large outbreaks of hepatitis E in developing countries [3]. In contrast, GT3 and GT4 are zoonotic and mostly transmitted to humans by direct contact with an infected animal or by consumption of undercooked meat products from an infected animal [1]. These GTs are the main cause of sporadic hepatitis E in industrialized countries. In Europe, subtypes 3c, 3e and 3f are predominant in hepatitis E patients [5].

The main animal reservoir for GT3, besides domestic pigs, are wild boars [6]. Several case reports describe human disease after consumption of wild boar liver or meat [7,8]. Worldwide, mean RNA prevalence of 0–68.2% and mean antibody prevalence of 1.6–57.4% are reported for wild boars [6]. In addition, the zoonotic HEV GT4 as well as GT5 and GT6, which have not yet been detected in humans, have been described in wild boars [9].

Despite the growing molecular and serological evidence of wild boar infection with zoonotic HEV strains, direct proof of infectivity by virus isolation from wild boar samples has only scarcely been reported so far. The main reason is the general difficulty to isolate HEV strains in cell culture [10]. If successful, the virus grows very slowly without any cytopathic effect. Most of the established culture-adapted HEV strains have been isolated from chronically infected human patients; those strains often contain genome insertions in their hypervariable region (HVR) of ORF1 [11]. These insertions in the HVR are associated with a growth advantage [12,13]. However, recent optimization of culture protocols using the human liver carcinoma cell line PLC/PRF/5 allowed the isolation of several human HEV strains without genome insertions directly from clinical samples [14]. In contrast, successful isolation of domestic pig-derived HEV wildtype strains required the use of a sophisticated 3D PLC/PRF/5 culture system [15,16]. HEV cell culture-adapted strains derived from wild boars are rarely reported and have been mainly generated using reverse genetics approaches [17].

To extend the understanding of zoonotic HEV strains using the cell culture approach, this study was designed to isolate HEV in cell culture directly from wild boar samples. To this end, a panel of pre-selected HEV positive wild boar liver and serum samples was inoculated onto PLC/PRF/5 cells and incubated according to a previously described, optimized culturing protocol [14]. Virus growth was monitored using RT-qPCR, immunofluorescence and electron microscopy. The complete genome sequences of two successfully isolated strains were generated using a newly developed NGS method, indicating that one of the strains belongs to subtype 3f and the other to a putative new subtype. No insertion was present in the HVR of these strains. Although the newly isolated strains showed slow growth on PLC/PRF/5 cells, they may be useful in future studies, e.g., by assessing their specificity to different hosts and cell-types, or by comparing their characteristics with other cell culture-adapted HEV strains derived from human patients.

## 2. Materials and Methods

### 2.1. Wild Boar Samples

Five serum samples and five liver samples were selected from a wild boar sample collection derived from an HEV prevalence study in wild boars from Germany. The samples originated from free-ranging populations living in military training areas of the German armed forces. Details on the samples are presented in Table 1. Samples were collected directly after hunting and were stored at −80 °C until use.

### 2.2. Detection and Quantification of the HEV Genome

RNA was extracted from liver samples as described [18] using the RNeasy mini kit (Qiagen, Hilden, Germany). RNA from serum and cell culture supernatants was extracted using the NucliSens^®^ EasyMag^®^ system (Biomérieux, Nürtingen, Germany). HEV-RNA was detected using primers and probe as previously described [19] and the QuantiTect Probe RT-PCR kit (Qiagen). Quantification of HEV copy numbers was accomplished by using an external standard of in vitro-transcribed HEV-RNA as previously described [20].

### 2.3. Genotyping

The RNA was subjected to RT-PCR and nested PCR according to Johne et al. [21], amplifying a 280 bp fragment (excluding primer sequences) of the RNA-dependent RNA polymerase (RdRp) region of ORF1. The PCR products were Sanger sequenced by a commercial provider (Eurofins Genomics Germany GmbH, Ebersberg, Germany) and the resulting sequences were used for genotyping with the Hepatitis E Virus Genotyping Tool Version 0.1 (https://www.rivm.nl/mpf/typingtool/hev/, accessed on 4 September 2021).

### 2.4. Isolation of HEV in Cell Culture

A total of 1 g of each liver sample was homogenized in 1 mL PBS using the FastPrep^®^-24 homogenizer (MP Biomedicals, Irvine, CA, USA) for 2 × 30 s in 50 mL tubes with 3 ceramic beads (“1/4” Ceramic Sphere, MP Biomedicals) added. Serum samples (50 µL each) were diluted in 450 µL PBS containing 0.2% BSA. The homogenized liver samples and the diluted sera were centrifuged at 6000× *g* for 10 min and the supernatant sterile filtrated using a 0.2 µm PES membrane (Millex GP 0.22 µm, Merck Millipore, Darmstadt, Germany) before use as inoculum. Cell culture supernatant containing the cell culture-adapted genotype 3c strain 47832c [22,23] was used as a control. Cell culture infection trials were performed based on a protocol published by Schemmerer et al. [14]. Briefly, PLC/PRF/5 cells were seeded with a concentration of 10^5^ cells/mL into a T25 flask using 5 mL MEMM (MEM, 10% FCS, 2 mM glutamine, 1% NEAA, penicillin/streptomycin, 2.5 µg/mL amphotericin B and 30 mM MgCl_2;_ PAN Biotech, Aidenbach, Germany). Cells were maintained at 37 °C and 5% CO_2_ for two weeks, while exchanging the entire media every 3 to 4 days. For cell infection, the medium was removed, 500 µL inoculum was added and incubated at room temperature for 75 min. Thereafter, 5 mL of MEMM was added, without removing the inoculum, and cells were incubated at 34.5 °C and 5% CO_2_ for 1 day. The medium was completely changed every 3 to 4 days until the end of the experiment. Cell culture supernatant samples were taken during medium exchanges and stored at −20 °C. The first virus passage was finished at 3 months (96 days post-infection, d p.i.) after inoculation by storing the complete supernatant at −20 °C. A second virus passage, using 500 µL of the final cell culture supernatant from the first passage, was continued for 6 weeks as described for the first passage.

### 2.5. Immune Electron Microscopy

The complete method used for immune electron microscopy is described by Horvatits et al. [24]. Briefly, 10 µL of each of the samples was adsorbed onto carbon/formvar -oated copper grids (Plano GmbH, Wetzlar, Germany) and contrasted with 2% uranyl acetate. Detergent treatment was performed before adsorption with 0.1% sodium deoxycholate (Na-DOC) for 10 min at 4 °C as described [25], to either remove the quasi envelope or to eliminate the virus-like particles of hepatitis B virus surface antigen. For immune-detection, an HEV capsid protein-specific monoclonal antibody [26] was used together with a gold-labeled secondary antibody. The samples were examined using a JEM 1400 transmission electron microscope (JEOL GmbH, Freising, Germany) operated at 120 kV. Imaging was performed using a Veleta G2 camera (EMSIS GmbH, Münster, Germany). Particles size measurement was done using ITEM software (Olympus, Hamburg, Germany).

### 2.6. Immunofluorescence

PLC/PRF/5 cells were seeded in 96 well plates and inoculated 2 weeks later with 100 µL of a 10-fold dilution series of supernatants from 6 weeks (49 d) p.i. of the second passage according to the infection protocol as described above. At 2 weeks after infection, the medium was removed and cells were fixed using acetone/methanol (1:1) for 30 min at 4 °C. Fixed cells were washed with PBS and blocked with PBS containing 1% FCS for 1 h at 37 °C. The solution was removed and a 1:500 dilution of an HEV capsid protein-specific rabbit hyperimmune serum [23] in PBS containing 1% FCS was added. After 1 h incubation at 37 °C, the antibody was removed and cells were washed three times with PBS. Thereafter, a 1:1000 dilution of FITC-conjugated anti-rabbit IgG (Sigma, Deisenhofen, Germany) in PBS containing 1% FCS was added and the cells were incubated for 1 h at 37 °C. The secondary antibody was removed and the cells were washed twice with PBS and once with distilled water. The cells were mounted with Roti^®^-Mount FluorCare DAPI (Carl Roth, Karlsruhe, Germany) and analyzed using an Axio Observer Z1 microscope (Carl Zeiss, Oberkochen, Germany).

### 2.7. Sequencing of the Hypervariable Region (HVR)

The HVR was amplified using RNA extracted from the original material of all inoculated samples, from the second passage of the two isolated strains and from the positive control virus. For amplification, an RT-PCR was performed using primers HVR-s (5′-TGG TCT ACA TCT GGY TTY TCT AG-3′) and HVR-as (5′-GGA TTT GAC GCR TTN ACC AGC CA-3′), which were delineated from an alignment of different HEV GT 3 subtype sequences and resulted in a 343 bp PCR product for most strains (without HVR insertions). RT-PCR was performed using the One-Step RT-PCR kit (Qiagen). The temperature profile included reverse transcription at 42 °C for 30 min, enzyme activation at 95 °C for 15 min, followed by 40 cycles with denaturation at 94 °C for 30 s, annealing at 55 °C for 30 s and elongation at 74 °C for 45 s, followed by a final elongation at 74 °C for 5 min. PCR products were Sanger sequenced using a commercial provider (Eurofins).

### 2.8. Whole Genome Sequencing of HEV Strains

Nucleic acids were extracted from cell culture supernatants of the second passage from 49 d p.i using the automated extraction platform EMAG and NUCLISENS^®^ EASY-MAG^®^ reagents (bioMérieux, Marcy-l’Étoile, France). Libraries were generated using the KAPA RNA HyperPrep Kit for Illumina^®^ platforms (Roche Diagnostics, Mannheim, Germany) following the manufacturer’s instructions. Settings that were to be specified individually in the manufacturer’s instructions during library preparation were chosen as follows. For RNA fragmentation, the setting 85 °C for 6 min was used. Library amplification using the KAPA Hifi HotStart Ready Mix (2x, Roche Diagnostics) was accomplished in 10 amplification cycles (chosen based on starting RNA amounts of 30 ng and 40 ng RNA for Wb108/17 and Wb257/17, respectively) using P5 and P7 primers provided with the Kit. The size distribution of the final library was monitored on the Fragment Analyzer 5200 (Agilent Technologies, Santa Clara, CA, USA) using the Agilent DNF-474 HS NGS Kit (Agilent Technologies) and library concentration was determined using the Qubit HS dsDNA Assay Kit (Fisher Scientific, Suwanee, GA, USA). HEV sequences were subsequently enriched using the NGS-library target enrichment system myBaits^®^ (Arbor Bioscience, Ann Arbor, MI, USA obtained from BioCat, Heidelberg, Germany).

The myBaits^®^ set used here for specific enrichment of HEV from NGS libraries was custom-designed by author V.M.C. to cover all Orthohepeviruses. Briefly, an alignment of 346 sequences of *Orthohepevirus A* to *D* (approximately 1.2 megabases) was used. After the exclusion of the HVR of HEV, 80 nt baits were designed with ~1.6-fold tiling density, resulting in 22,924 candidate baits. To increase later performance of the bait set, the number of baits was reduced by using default in-silico analyses provided by the company (Arbor Bioscience, Ann Arbor, MI, USA). These comprised omitting low-complexity repeats, clustering of baits with more than 95% identity, and blast filtering against human, rat, and pig genomes to exclude enrichment of host sequences, and removing all baits with more than 70% GC content. The final bait set contained 17,165 baits (sequences available upon request).

HEV sequence enrichment from the library was done following the manufacturer’s recommendation or were chosen as follows. The libraries of both samples were combined and HEV sequences enriched in one capture reaction. Hybridization was accomplished at 65 °C for 18 h (overnight) and the temperature of 65 °C was used for all consecutive hybridization washing steps. After elution from streptavidin beads at 95 °C for 5 min, the enriched library was amplified in 20 cycles using the KAPA Hifi HotStart Ready Mix (2x, Roche Diagnostics) and P5/P7 primers obtained from integrated DNA technologies (IDT, Coralville, IA, USA). The enriched library was purified post amplification using the Monarch PCR & DNA Cleanup Kit (New England Biolabs Inc, Ipswich, MA, USA), pooled with 118 additional libraries and paired-end sequenced on the NextSeq 500 Sequencer (Illumina, San Diego, CA, USA) using the NextSeq 500/550 Mid Output Kit v2.5 (300 cycles) (Illumina).

### 2.9. Sequence Assembly and Phylogenetic Analysis

All analyses of NextSeq sequencing data were done using the software Geneious Prime^®^ 2020.2.2 (Biomatters Ltd. Auckland, New Zealand) with default parameters. For exclusion of short reads and trimming, the BBDuk Plug-In of Geneious Prime^®^ was used. HVR sequences derived by Sanger sequencing (see Section 2.7) were manually trimmed by deletion of primer sequences using the SeqBuilder Software (Lasergene, Madison, WI, USA). Mapping of the trimmed NexSeq reads was performed in Geneious Prime^®^ using reference sequences from a list with all HEV GT3 subtype reference strains [27]. All reads from NextSeq sequencing assigned to the reference sequences and the HVR sequences were assembled de novo for each sample individually using Geneious Prime^®^ to obtain the whole genome sequences. The sequences were annotated by transferring annotations from the closest relating HEV GT3 subtype reference sequence [27] using Geneious Prime^®^ and submitted to GenBank. Phylogenetic trees were constructed with the neighbor-joining method and 1000 bootstrap iterations using the Tree Builder module in Geneious Prime^®^ 2020.2.2 after nucleotide alignment using MUSCLE.

## 3. Results

### 3.1. Selection of Samples, HEV Quantification and Typing

**Figure 1:**
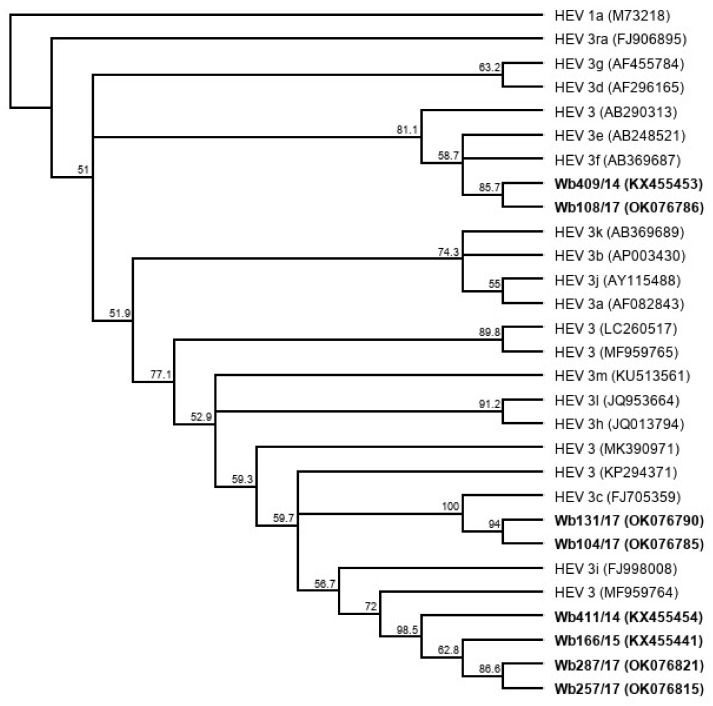
Cladistic relationship of sequences from wild boar samples (highlighted in bold) with proposed HEV GT3 subtype reference sequences [27]. The tree was constructed from a MUSCLE nucleotide alignment of a 280 nucleotide fragment of the RNA-dependent RNA polymerase (RdRp) region using the neighbor-joining method and 1000 bootstrap iterations, and rooted with HEV 1a. Bootstrap values >50% are shown.

Five liver samples and five serum samples were selected from a sample collection of HEV-positive wild boar from Germany (Table 1). The liver samples contained between 9 × 10^7^ and 2 × 10^9^ HEV genome copies (gc)/mL, and the serum samples between 5 × 10^6^ and 5 × 10^7^ gc/mL. Using the HEV genotyping tool with a sequence fragment amplified from the RdRp region, the subtype 3c was assigned to three samples and subtype 3f to two samples. The remaining five samples could not be subtyped using the HEV genotyping tool. They showed the highest sequence identities with the wild boar strain WB/HEV/NA17ITA15 (GenBank acc.-no. MF959764), which is classified as a provisional new subtype according to Smith et al. [27]. A phylogenetic tree based on the sequence fragment of the RdRp region confirms the close relationship of the strains with the respective HEV GT3 subtype reference strains (Figure 1).

### 3.2. Isolation of HEV Strains in Cell Culture

**Figure 2:**
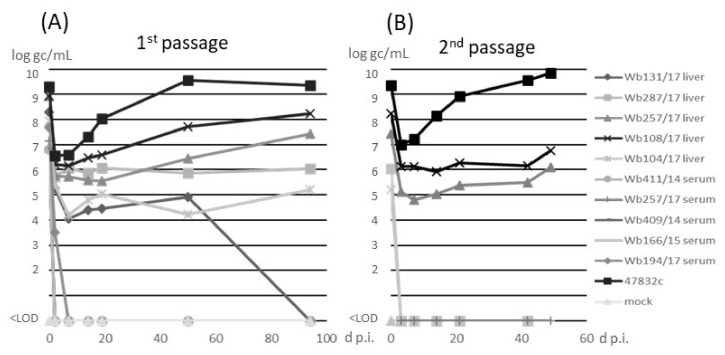
Detection of HEV RNA in cell culture supernatant after (**A**) inoculation of PLC/PRF/5 cells with wild boars samples (1st passage) or (**B**) inoculation of the supernatants from 96 days post-infection (d p.i.) of the 1st passage on fresh PLC/PRF/5 cells (2nd passage). Supernatants of first and second passage were analyzed by RT-qPCR and the amounts are shown in log genome copies (gc)/mL. <LOD, below limit of detection.

The samples were inoculated onto monolayer cultures of PLC/PRF/5 cells as previously described [14]. The cell culture-adapted strain 47832c was used as a control. No cytotoxic effects were recorded after inoculation of the samples. Analysis of culture supernatants for the presence of HEV RNA by RT-qPCR (Figure 2A) indicated an initial decline of the RNA at 3 d p.i. for all samples. For serum samples, no HEV RNA could be detected after 3 d p.i. In contrast, all liver samples showed constant or slowly increasing amounts of HEV RNA between 3 d p.i. and 50 d p.i. With the exception of sample w131/17, for which HEV-RNA could not be detected after 50 d p.i., all wild boar samples showed slightly increasing HEV RNA amounts until 94 d p.i., when the first passage was finished. The HEV gc numbers determined from cell culture supernatant of virus isolations from the different wild boar liver samples ranged from 1 × 10^5^ and 2 × 10^8^ gc/mL, whereas 2 × 10^9^ gc/mL were determined for the cell culture-adapted 47832c control strain, at 94 d p.i. of passage 1. In the second passage (Figure 2B), a decline of HEV RNA at 3 d p.i., similar as in passage 1, was evident (Figure 2A). At the following time-points, HEV RNA could only be detected in liver samples Wb108/17 and Wb257/17, which slightly increased to 6 × 10^6^ and 1 × 10^6^ gc/mL, respectively, until the end of the experiment at 49 d p.i. No cytopathic effects were observed in all samples over the whole time of the experiment.

### 3.3. Sequence Comparison of the HVR

**Figure 3:**
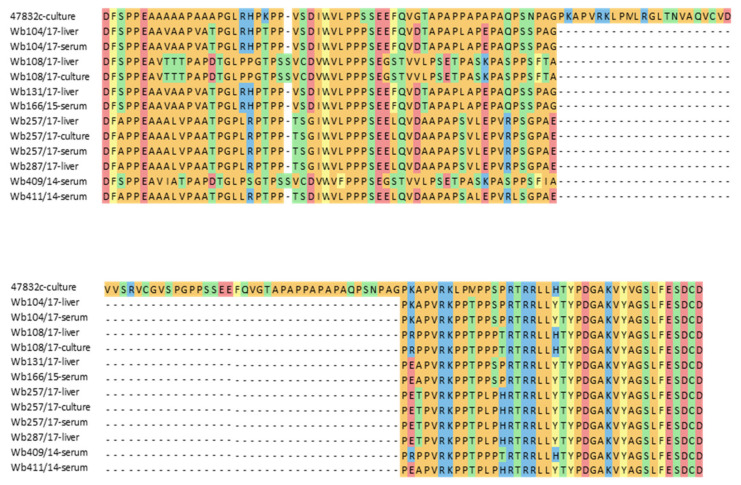
Alignment of amino acid sequences deduced from RT-PCR products of the hypervariable region (HVR) of the HEV genome. Sequences of the cell culture-adapted control strain 47832c containing a well-known insertion within the HVR (47832c-culture), wild boar serum and liver samples used for the cell culture isolation experiment (indicated as serum or liver), and cell culture-isolated HEV strains from samples Wb108/17 and Wb257/17 at 49 d p.i. of 2nd passage (indicated as culture) are shown.

Many HEV strains successfully isolated in cell culture in the past contain insertions within the HVR of their genomes. To test if differences in the HVR sequences of the used HEV strains might explain the differences in successful cell culture isolation, a fragment of the HVR genome region was amplified from original material of all inoculated samples, from the second passage of the two isolated strains and from the positive control virus. Sequence analysis confirmed the presence of a large insertion in the cell culture-adapted control strain 47832c, but not in the inoculated samples or the two new cell culture isolates. A considerable degree of sequence heterogeneity is evident between the strains, also when the deduced amino acid sequences are compared (Figure 3). The HEV genotype 3f strains from samples w409/14 and Wb108/17 as well as the isolated strain from the latter sample contained an additional amino acid codon compared to the other strains.

### 3.4. Characterization of Isolated HEV Strains by Electron Microscopy and Immmunofluorescence

**Figure 4:**
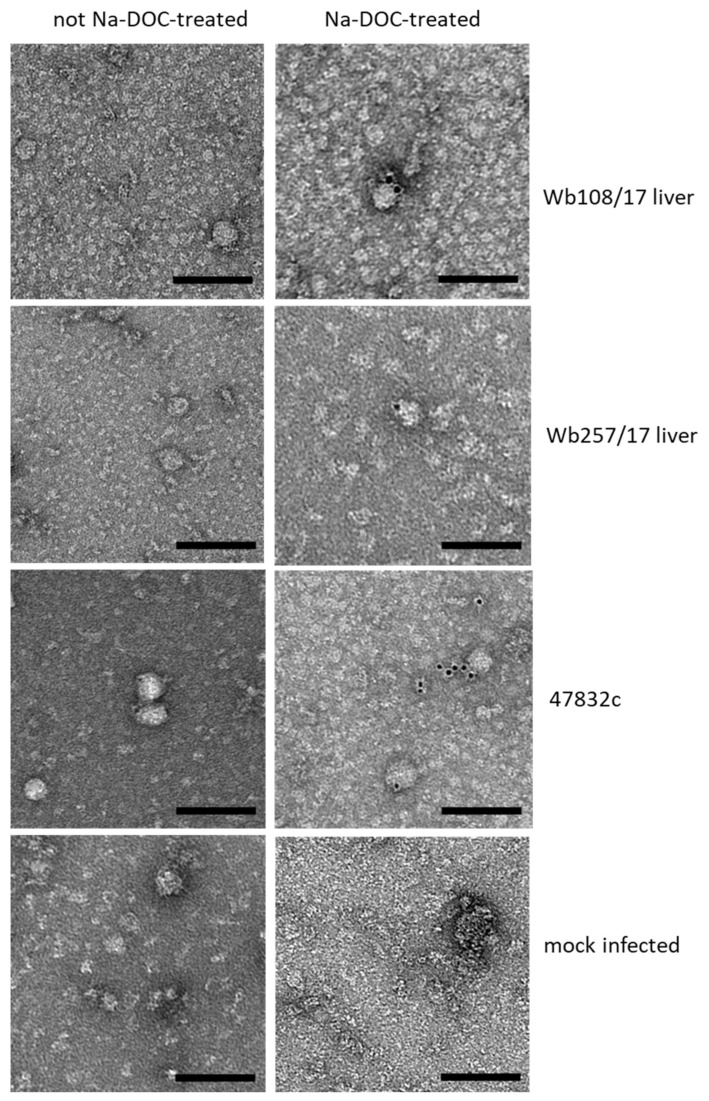
Analysis of particle morphology by immunogold staining and transmission electron microscopy. Samples, as indicated on the right side of the images per row, were treated (right image) with sodium deoxycholate (Na-DOC) to remove the quasi-envelop from HEV, or not treated (left image). Immunogold-staining was done using an anti-HEV capsid protein-specific monoclonal antibody together with a gold-labeled secondary antibody. Black dots represent the gold particles. Negative staining with uranyl acetate. Scale bar: 100 nm.

The supernatant aliquots from 49 d p.i. of the second passage were analyzed by immune electron microscopy. However, virus particle-like structures with diameters of 30–50 nm were found in all samples, including the negative control (Figure 4, bottom, left). As it is known that PLC/PRF/5 cells contain a hepatitis B virus (HBV) genome and can secrete HBV S antigen-containing virus-like particles of this size, further efforts were made to discriminate these structures from HEV particles. Treatment of the samples with sodium deoxycholate (Na-DOC) led to the absence of particle-like structures in the negative control (Figure 4 bottom, right), indicating that the HBV S antigen-containing particles have been eliminated through solubilization. Immunogold staining after treatment with Na-DOC using an antibody against the HEV capsid protein identified many gold-labeled particles with a diameter of approximately 40 nm in the positive control and fewer gold-labeled particles of similar shape in samples Wb108/17 and Wb257/17 (Figure 4, right). The particle shape was similar to that observed for cell culture-derived HEV in a previous study [25].

**Figure 5:**
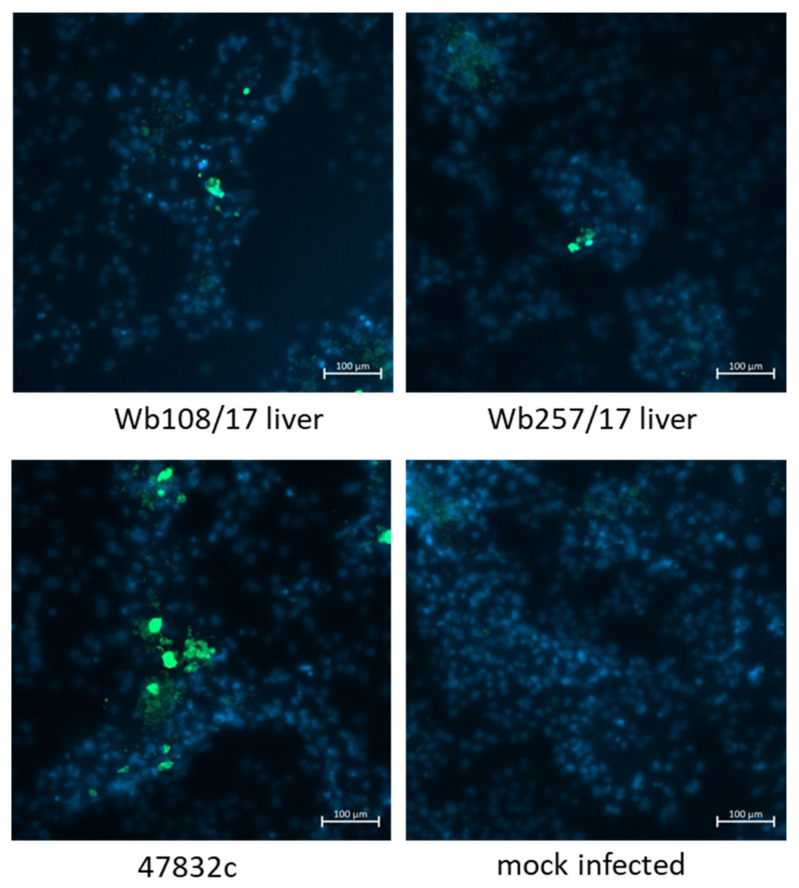
Immunofluorescence analysis of PLC/PRF/5 cells 2 weeks after infection with cell culture supernatants from 49 d p.i. of the 2nd passage. Staining was done using an anti-HEV capsid protein-specific antiserum (green staining). Cell nuclei were stained with DAPI (blue staining). Scale bar: 100 µm.

Virus-infected cells were further analyzed by immunofluorescence using an antiserum against the HEV capsid protein. To this end, the culture supernatants from 49 d p.i. of the second passage were inoculated onto fresh PLC/PRF/5 cells and analyzed at 2 weeks after infection. As evident from Figure 5, only a few stained cells could be demonstrated for samples Wb108/17 and Wb257/17 in the wells inoculated with the undiluted samples, whereas more stained cells were present in the positive control. No HEV-specific staining was found in the negative control.

### 3.5. Genome Sequencing and Phylogenetic Analysis of Isolated HEV Strains

Next generation sequencing of HEV sequence-enriched libraries was performed for the two isolated HEV strains using RNA extracted from cell culture supernatants of the second passage (from 49 d p.i.). The sequencing run of sample Wb108/17 produced a total number of 3,305,130 reads and that of sample Wb257/17 a total of 4,250,116 reads. To identify HEV reads, all reads per sample were mapped against a list of the reference sequences for HEV GT 3, as proposed by Smith et al. [27]. By this, 666,278 reads of sample Wb108/17 were assigned to the HEV GT3 subtype reference sequences and assembled de novo, producing a unique contig of 7578 nt from 128,359 reads. The sequence was manually trimmed to 7219 nt post annotation and submitted to Genbank (GenBank acc.-no. OK076715). For sample Wb257/17, 101,163 reads were assigned to the HEV GT3 subtype reference sequences and assembled de novo, producing a unique contig of 7383 nt from 14,847 reads. The sequence was manually trimmed to 7215 nt post annotation and submitted to Genbank (GenBank acc.-no. OK076716). Both sequences represented complete HEV genomes. Genome coverage for Wb108/17 was between 475 and 6099 with a mean coverage of 2572. The genome coverage for Wb257/17 ranged between 6 to 819, with a mean coverage of 300. The coverage was particularly low in the HVR region of both strains, because the bait design did not cover this region.

**Figure 6:**
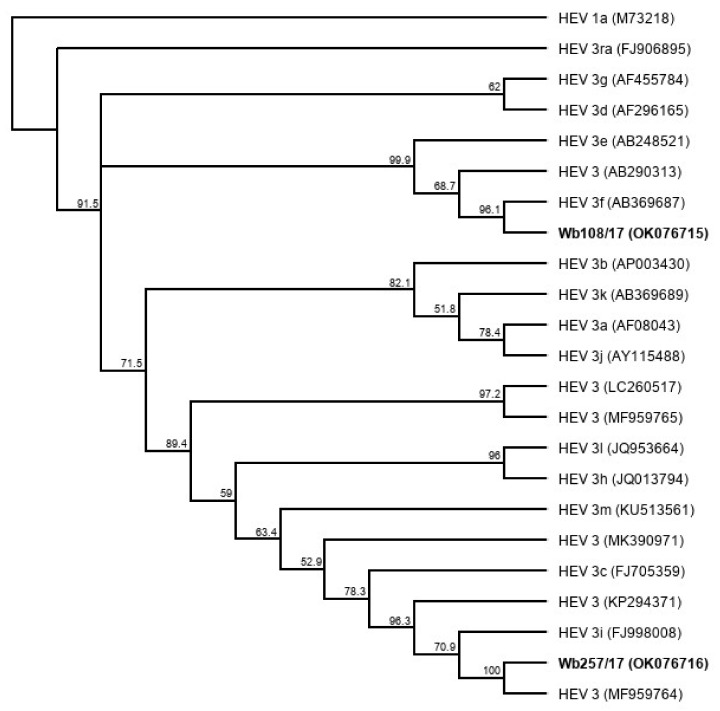
Cladistic relationship of cell culture-isolated whole genome sequences of HEV from wild boar samples Wb108/17and, Wb257/17 (highlighted in bold) and proposed HEV GT3 subtype reference sequences [27]. The tree was constructed from a MUSCLE nucleotide alignment using the neighbor-joining method and 1000 bootstrap iterations and rooted with HEV 1a. Bootstrap values >50% are shown.

A phylogenetic tree based on the complete genome sequences of the newly isolated HEV strains (Figure 6) shows a close relationship (91.7% genome identity in pairwise alignment) of Wb257/17 with an HEV 3 reference strain (GenBank acc.-no. MF959764), which has not yet been assigned a subtype [27]. The HEV isolate Wb108/17 clustered with the HEV subtype 3f reference strain (GenBank acc.-no. AB369687) (87.5% genome identity in pairwise alignment).

## 4. Discussion

Isolation of HEV in cell culture is generally difficult and time-consuming, and only a few strains efficiently replicating in cell culture have been described so far [11]. Although HEV GT3 is a zoonotic virus, most of the previously isolated strains originate from human patients [11], whereas HEV strains derived from wild boars have only rarely been described [28,29]. Here, we isolated two HEV strains from wild boar and characterized them further in order to elucidate the prerequisites for successful isolation.

We used an established protocol, which was previously optimized and has proven to isolate different GT3 subtypes derived from human patients [14]. Successful propagation of several HEV genotypes including GT3, GT4, GT5 as well as ratHEV (which belongs to the species *Orthohepevirus C*) in PLC/PRF/5 cells, which were also used in this study, has already been described [14,30,31,32]. In addition, PLC/PRF/5 cells grown in 3D architecture led to successful isolation of HEV GT3 from a sausage sample containing pig liver [16]. This cell line is derived from a human liver carcinoma and produces hepatitis B virus surface antigen [33]. Although the possible co-infection with hepatitis B virus might be problematic for studying some aspects of the HEV life cycle, the cell line should be considered well suited for initial isolation of HEV from field samples. This was confirmed in our study showing its successful use for HEV isolation from wild boar liver samples. The successful isolation also indicates that the applied HEV isolation protocol, originally developed for human serum, plasma and fecal samples [14], is suitable for a wide range of sample types including liver homogenates from animals and can therefore be broadly used in the future.

Virus isolation was only successful for two out of ten inoculated samples. As two different HEV subtypes were successfully isolated, the subtype does not seem to generally restrict virus infection of the cells. When liver and serum from the same animal (Wb257/17) were inoculated, virus could only be isolated from the liver, thus arguing against strain specificity as the major restriction factor. This observation could however suggest that the sample type is an important factor for HEV cell culture propagation, as successful HEV isolation was accomplished from liver samples, but not from serum. In contrast, several human HEV strains have been successfully isolated from patient serum indicating that serum generally is an appropriate sample type for HEV isolation in cell culture [22,34]. One major difference between liver and serum samples in our study was their amount of HEV RNA, which was generally higher in liver samples compared to serum samples. Moreover, virus isolation was only successful for two samples showing very high HEV RNA amounts (8.66 × 10^8^ and 8.48 × 10^8^ gc/mL). Other studies have also shown a strong correlation between successful HEV isolation and a high amount of virus in samples [28,34]. The successful HEV isolations from human serum may therefore be attributed to high virus amounts in the serum of human patients, which are rarely found in wild boars.

In cell culture, the two isolated strains showed a permanent HEV RNA production and could be passaged on the cell cultures, thus indicating a robust infection of the PLC/PRF/5 cells. However, compared to the cell culture-adapted strain 47832c, growth was slow and the reached endpoint titers were low. In addition, only a few cells showed strong fluorescence and lower particle numbers were identified by electron microscopy, indicating a lower production of viral antigen and virus particles as compared to strain 47832c. This strain contains a genome insertion in its HVR derived from two duplications of its genome, which has been recently shown to be essential for its highly efficient replication in cell culture [13]. An essential role of insertions in the HVR for highly efficient replication in cell culture has also been shown for other HEV strains [12,35]. Sequencing of the HVR of samples Wb257/17 and Wb108/17 demonstrated the absence of insertions in the original samples as well as in the cell culture-passaged supernatants, in line with their slow growth. Although the slow growth may be a disadvantage for broad use of the strains in research, it should be considered that strains without insertions might better resemble wild-type strains compared to those with insertion.

Analysis of the particle morphology using electron microscopy indicated spherical structures, which could be labeled by immunogold staining with an HEV capsid antibody only after treatment with sodium deoxycholate. It has been shown that this treatment removes the envelope from HEV particles, thus making the capsid protein assessable for antibodies [24]. Therefore, it has to be concluded that cells released mainly quasi-enveloped particles into the supernatant after infection with the HEV strains from wild boar. This is in accordance with findings for other HEV strains in cell culture [4,25]. The presence of particle structures similar to HEV in the untreated non-infected culture supernatant, which disappeared after sodium deoxycholate treatment, further complicated the structural analyses. Although the origin of these structures has not been identified, they may represent hepatitis B virus-like particles as the presence of the hepatitis B virus surface antigen has been demonstrated in the supernatant of PLC/PRF/5 cells [33].

The whole genomes of the isolated strains were obtained by next generation sequencing, using a newly developed HEV-specific post library-preparation sequence enrichment technique. The technique allowed sequencing of the complete HEV genomes with high mean sequence coverages, showing its suitability to cell culture supernatants. The enrichment technology separates HEV sequences from other sequences present in the sample library prior to sequencing and the principle has been previously successfully used for other viruses [36,37]. Further investigations will show whether this method is just as suitable for sample matrices other than cell culture supernatant, which contain higher amounts of non-HEV RNA.

Genotyping and whole genome sequence comparison to HEV subtype reference strains confirmed that strain Wb108/17 belongs to subtype 3f. This subtype has been previously identified in samples from humans, animals, food and the environment in Germany [38,39,40,41], also representing one of the major subtypes in Europe [5]. Recently, a subtype 3f-like strain from a human patient has also been isolated in cell culture [14]. Comparison of the human-derived strain with those of the wild boars is of interest for future investigation on the zoonotic characteristics of this subtype. Typing of strain Wb257/17 was more complicated as it did not cluster with established subtypes but was closely related to a recently identified wild boar strain from Italy [42]. Smith et al. [27] grouped this strain as a potentially new subtype but stated that more full-length genome sequences of epidemiologically unrelated strains are necessary to establish a new subtype [27]. The genome sequence generated in our study confirms the continued presence of the new subtype and may be helpful for its official classification in the future.

## 5. Conclusions

In conclusion, the isolation of two HEV strains from wild boar samples in cell culture was successful. The applied protocol has been proven to be suitable for wild boar liver samples in addition to already described human serum, plasma and fecal samples, therefore it might be used for a broader range of sample types and sources in the future. The isolated strains were generally characterized based on their growth kinetics, particle morphology and whole genome sequence. Subtyping indicated the isolation of a zoonotic subtype 3f strain and of a putative novel subtype. Both isolates are now available for further basic and applied research as well as for comparison with other animal- and human-derived HEV strains.

## Figures and Tables

**Table 1:** Wild boar samples from Germany used in the study and initial hepatitis E virus (HEV) RNA concentration for each sample. The two samples, from which HEV was successfully isolated by passaging on PLC/PRF/5 cells are indicated in bold face.

Sample Number	Sample Type	Year	HEV RNA (gc/mL)	HEV Subtype
Wb104/17 ^1^	liver	2017	9.00 × 10^7^	3c
Wb104/17 ^1^	serum	2017	4.94 × 10^7^	3c
**Wb108/17**	**liver**	**2017**	**8.66 × 10^8^**	**3f**
Wb131/17	liver	2017	1.98 × 10^8^	3c
Wb166/15	serum	2015	5.24 × 10^6^	3^3^
**Wb257/17 ^2^**	**liver**	**2017**	**8.48 × 10^8^**	**3^3^**
Wb257/17 ^2^	serum	2017	1.39 × 10^7^	3^3^
Wb287/17	liver	2017	2.08 × 10^9^	3^3^
Wb409/14	serum	2014	7.26 × 10^6^	3f
Wb411/14	serum	2014	6.62 × 10^6^	3^3^

^1,2^ liver and serum sample from the same animal. ^3^ not typed by the HEV genotyping tool.

## Data Availability

Data are available upon request by R.J. (Reimar.johne@bfr.bund.de). Bait sequences used for HEV genome sequencing are available upon request by V.M.C. (victor.corman@charite.de).
